# High-Throughput Method for Detection of Arbovirus Infection of Saliva in Mosquitoes *Aedes aegypti* and *Ae. albopictus*

**DOI:** 10.3390/v12111343

**Published:** 2020-11-23

**Authors:** Nildimar Alves Honório, Daniel Cardoso Portela Câmara, Keenan Wiggins, Bradley Eastmond, Barry Wilmer Alto

**Affiliations:** 1 Laboratório de Mosquitos Transmissores de Hematozoários, Instituto Oswaldo Cruz, Fundaҫão Oswaldo Cruz, 21040-360 Rio de Janeiro, Brazil; dcpchamber@gmail.com; 2 Núcleo Operacional Sentinela de Mosquitos Vetores-Nosmove/Fiocruz, Fundaҫão Oswaldo Cruz, 21040-360 Rio de Janeiro, Brazil; 3 IFAS, Department of Entomology and Nematology, Florida Medical Entomology Laboratory, University of Florida, Vero Beach, FL 32962, USA; keenan.wiggins@gmail.com (K.W.); b-eastmond@hotmail.com (B.E.); bwalto@ufl.edu (B.W.A.)

**Keywords:** mosquito vectors, vector competence, saliva assay, transmission, arbovirus surveillance

## Abstract

Vector competence refers to the ability of a vector to acquire, maintain, and transmit a pathogen. Collecting mosquito saliva in medium-filled capillary tubes has become the standard for approximating arbovirus transmission. However, this method is time-consuming and labor-intensive. Here we compare the capillary tube method to an alternative high-throughput detection method the collection of saliva on paper cards saturated with honey, with (FTA card) and without (filter paper) reagents for the preservation of nucleic acid for *Aedes aegypti* and *Aedes albopictus* mosquitoes infected with two emerging genotypes of the chikungunya virus (CHIKV). Model results showed that the Asian genotype CHIKV dissemination in the harvested legs of both *Ae. aegypti* and *Ae. albopictus* increased the odds of females having a positive salivary infection and higher salivary viral titers, while for the IOL genotype the same effect was observed only for *Ae. aegypti*. Of the three tested detection methods, the FTA card was significantly more effective at detecting infected saliva of *Ae. aegypti* and *Ae. albopictus* females than the capillary tube and filter paper was as effective as the capillary tube for the Asian genotype. We did not find significant effects of the detection method in detecting higher viral titer for both Asian and IOL genotypes. Our results are discussed in light of the limitations of the different tested detection methods.

## 1. Introduction

Invasive mosquitoes *Aedes aegypti* and *Ae. albopictus* are regarded as the main transmitters of emerging arboviruses affecting human health. Zika, chikungunya, and dengue viruses are widespread worldwide, pose a significant public health threat, and are transmitted primarily by *Ae. aegypti* and *Ae. albopictus*. The chikungunya virus emerged in Kenya in 2004 (Eastern/Central/Southern African, ECSA, lineage) and La Reunion in 2005 to 2006 (Indian Ocean lineage) causing outbreaks of chikungunya fever [1,2,3]. Local transmission of chikungunya virus was first detected in the Caribbean in 2013, followed by spread throughout the Americas by 2015 (Asian lineage, and ECSA lineage in Brazil) [4,5,6]. Zika virus is comprised of an Asian lineage and two African lineages [7]. The Asian lineage of Zika virus first emerged in Micronesia in 2007, followed by an outbreak in French Polynesia in 2013 [8] and Brazil in 2015 [9]. Following arrival, the Zika virus quickly spread throughout the Americas causing human infection. Zika and chikungunya transmission frequently occur in regions already coping with the public health burden of dengue endemicity.

Vector competence is an entomological parameter that refers to the intrinsic ability to transmit a pathogen, including susceptibility to infection, propagation and/or development, and transmission. Vector competence is a parameter of vectorial capacity which determines the relative importance of a mosquito as a vector. Vector competence is one of several parameters, some of which contribute more strongly, that determine the risk of disease transmission [10]. The ingestion of an arbovirus infectious bloodmeal elicits immune and transcriptional responses in the mosquito [11] and subsequent gene products interact with the environment and, in part, determine vector competence. Arboviruses must overcome barriers to infection to propagate and allow for transmission to a vertebrate host by bite. Following ingestion, the infected blood is deposited in the mosquito mesenteron (midgut) and the arbovirus infects midgut epithelial cells. The probability of an arbovirus infection of the midgut is often dose-dependent, and failure to establish a localized infection is attributable to the midgut infection barrier(s) [11]. Contributing factors that inhibit infection may be physical and innate immunity-related barriers, including RNA interference [12,13], serine proteases [14], midgut microbiota [15], symbionts (*Wolbachia*, [16], and refractoriness of midgut epithelial cells to infection [11,17]. Propagation of the arbovirus in the midgut is followed by entry into the hemocoel and dissemination to secondary tissues (e.g., fat body, hemocytes, nerve tissue). A midgut escape barrier may prevent the arbovirus from spreading to other tissues [18]. Mosquitoes with a non-disseminated infection are incapable of transmitting the virus by bite. It is well-established that the proportion of mosquitoes with disseminated infection increases with time since the ingestion of infectious blood for those mosquitoes with a midgut infection. Following disseminated infection, the arbovirus population usually must propagate in tissues outside the midgut (secondary amplification) to facilitate further spread to the salivary glands [13]. A competent mosquito must cope with these barriers and establish an infection in the salivary glands (salivary gland infection barrier) and release the virus into salivary ducts (salivary gland escape barrier), allowing for virus-infected saliva to infect a vertebrate host during blood feeding. Virus and mosquito genetics along with the environment shape vector competence.

Arbovirus maintenance and transmission depend on the availability of competent arthropod vectors. Determination of competence is usually accomplished through laboratory infection studies that challenge mosquitoes with arboviruses accomplished by allowing the ingestion of infected blood and subsequent testing of mosquito tissues and organs, and the vertebrate host they are allowed to feed on, for arbovirus infection. Infection studies allow for the determination of the relative competency of mosquito species for select arboviruses, especially relevant in instances of geographic expansion of mosquitoes and arboviruses and the emergence of new genotypes of arboviruses. Infection studies also provide estimates (susceptibility to infection, disseminated infection, extrinsic incubation period, and transmission) under defined conditions that may be used to parameterize models of risk of arbovirus transmission. Further, information on the relative competency of mosquito species for select arboviruses can inform mosquito control strategies in targeting likely vectors of emerging pathogens.

A rigorous assessment of vector competence would involve challenging field-relevant populations of mosquitoes with oral ingestion of an arbovirus infectious blood meal, followed by an incubation period(s), and subsequent testing transmission for one or more time points after the anticipated extrinsic incubation period. Transmission may be measured by allowing individual mosquitoes to feed on vertebrate hosts and then testing the hosts at a later point for infection. Although this approach is comprehensive, there are several logistical constraints that make routine use of this method impractical. Mosquito infection studies often make use of hundreds or thousands of mosquitoes that would each need to be paired with a restrained vertebrate host in restricted biosafety level two and three conditions. The additional work needed to acquire and handle large numbers of vertebrates, as well as synchronizing feeding on hosts in single-day assays, makes it impractical to obtain numerous completed transmission tests. For these reasons, alternative approaches have been developed. Some investigations have used measures of other tissues indicative of disseminated infection (e.g., legs) as an approximation for transmission potential. However, this approach assumes negligible salivary gland infection and escape barriers, which is not a valid assumption in several instances (e.g., Venezuelan equine encephalitis virus, [19] CHIKV, [20,21]), thus overestimating transmission rates. Another approach is to dissect mosquito salivary glands and test them for arbovirus infection. However, there may be issues associated with this approach as well, including contamination of salivary glands with other tissues during dissection, and time constraints on assaying sufficient numbers of fresh specimens. Also, it is not a direct measure of saliva that is released from the salivary glands into salivary ducts during feeding.

For decades medical entomologists have been using a capillary tube method to capture saliva expectorates from live mosquitoes [22]. Briefly, capillary tubes are loaded with a dilute (media, fetal bovine serum, or mineral oil) and maintained at 37 °C. Within a glovebox, the wings and legs of a cold anesthetized mosquito are dissected, and the body is fastened to adhesive tape on a platform. With the aid of light microscopy, the proboscis of the mosquito is placed in the capillary tube and allowed to collect saliva expectorates for approximately 1 h. The contents of the capillary tube are then expelled in a centrifuge tube to be later tested by cell culture or molecular methods for the presence of arboviral infection or viral RNA. Shortcomings of this approach include the necessary use of several investigators to perform the activities, excessive handling of infected mosquito specimens, the lack of a visual, or molecular marker indicating that saliva was deposited in the capillary tube, and it is time-consuming.

A novel method has been developed to overcome several of these shortcomings. Twenty-four hours before measuring mosquitoes for transmission potential, mosquitoes were starved of sugar, but not water. Mosquitoes were individually placed in clear plastic tubes fitted with a removable screen lid (37 mL, 8 by 3 cm). The interior of each screen lid is fitted with a piece of filter paper (1 cm diameter) saturated with honey that has been dyed with blue food coloring (McCormick). Mosquitoes that fed on the honey deposit saliva and the blue food coloring were visualized in the crop with aid of a light source. Typically, mosquitoes were examined for blue coloring in their crop after 24 and 48 h, during the transmission assay, and filter papers were collected for testing. The mosquitoes were not destructively sampled and so the same mosquito may have been tested at multiple time points, allowing for estimates of the extrinsic incubation period (EIP) on a per capita basis. This improved approach allowed for new opportunities to investigate the genetics of EIP using individual mosquitoes, and individual-based models used to characterize pathogen transmission. The visual marker allows the investigator to select only those mosquitoes that fed on the honey to accurately measure transmission potential. Further, this approach is a high throughput method allowing for hundreds of mosquitoes capable of being processed in a few hours by a single person because minimal handling of mosquitoes is needed. The mosquitoes can be examined for a blue marking, recorded, and the entire tube with mosquito and paper may be placed in an ultralow freezer for later testing. That is, the mosquito never has to be directly handled during the transmission assay. On a related topic, a similar approach was utilized in mosquito traps for the surveillance of arboviruses in field settings where captured mosquitoes in a trap feed on honey saturated filter paper (or other substrate) which were then tested for arbovirus infection [23]. Variations of this approach have made use of FTA cards, Q-paper, and USTOP cards that include chemical reagents that inactivate and preserve nucleic acid [24,25].

The current study’s objective is to compare the relative efficacy of the capillary tube, filter paper and FTA card methods for the detection of arbovirus infection in mosquito saliva expectorate. We approach this study using an experimental design that allows for the collection and comparison of saliva expectorate from cohorts of the same individuals of mosquitoes using at least two of the methods at a time. We utilize a model system of two emergent genotypes of chikungunya virus and primary vectors *Ae. aegypti* and *Ae. albopictus*.

## 2. Materials and Methods

### 2.1. Mosquito Populations and Rearing

Two separate mosquito infection studies were performed. In the first experiment, we used mosquitoes from populations of *Ae. aegypti* and *Ae. albopictus* from local collections in Florida and *Ae. aegypti* from the Dominican Republic. The inclusion of the Dominican Republic strain of *Ae. aegypti* enabled us to compare Brazilian and Florida *Aedes* mosquito vectors to a separate vector population in the Caribbean associated with numerous cases of CHIKV in the 2014 outbreak. Mosquito larvae were collected from containers present in cemeteries and salvage yards. In the second experiment, we used mosquitoes of *Ae. aegypti* and *Ae. albopictus* from local collections of larvae from containers in Okeechobee and Key West, Florida, and eggs from oviposition traps in Rio de Janeiro and Macapá, Brazil during a routine entomological survey [26].

Larvae were reared to adulthood on a diet consisting of equal parts of brewer’s yeast and liver powder at 26 to 28 °C using methods described by Alto et al. [21]. Pupae were transferred to water-filled vials and sealed to capture newly eclosed adults. Mosquitoes identified by species were added to Bugdorm cages (Bioquip products, Ranco Dominguez, CA, USA) and provided with 10% sucrose solution and water through cotton wicks at 26 to 28 °C and a 14:10 h light:dark photo regime. Mosquitoes were provided with weekly blood meals to propagate eggs through access to bovine blood contained within hog casing members (Hemostat Laboratories, Dixon, CA, USA) heated to 37 °C using a water bath. Mosquitoes laid eggs on paper towels set inside water-filled cups held in the cages.

To initiate experiments, F_2–3_ generation eggs from field-collected parents were synchronously hatched in deoxygenated water prepared in an insulated vacuum container powered with an electronic pump as described by Zimler and Alto [27]. Newly hatched larvae were counted and placed in plastic photo trays (25 cm width, 30 cm length, 5 cm height; Richard MFG Co. Fernandina Beach, FL, USA) with 150 larvae and 900 mL of tap water and 0.4 g larval food. Supplemental food was added again at the same level 3 to 4 days later. Pupae were transferred to Bugdorm cages with 10% sucrose solution and water and newly eclosed males and females were held together to facilitate mating for 7 to 8 days. One day before feeding trials, mosquitoes were cold-anesthetized and the females were transferred to cylindrical cages (height by diameter, 10 cm by 10 cm, 50 females/cage) with a mesh screen lid.

### 2.2. Chikungunya Virus Isolates, Propagation, and Mosquito Infection

Mosquito infection studies used two emerging genotypes of chikungunya virus. A strain of chikungunya virus was obtained in December 2013 from an infected human in the British Virgin Islands (Asian lineage, GenBank accession: KJ451624). Another strain of chikungunya virus was obtained from an infected patient in 2006 returning to France from Reunion, identified as the Indian Ocean genotype of chikungunya virus responsible for the outbreak in the Indian Ocean region (IOL, LR2006-OPY1, GenBank accession: KT449801). Virus stocks were created by propagating the chikungunya virus strains in cell culture using African green monkey kidney (Vero) cells, and subsequent viral titration by plaque assay. To prepare fresh virus for mosquito infection, confluent monolayers of Vero cells (175 cm^2^) were each inoculated with 500 µL dilute stock virus at a multiplicity of infection of 0.1 and incubated for 1 h at 37 °C and 5% CO_2_ atmosphere, after which 24 mL media (M199 medium supplemented with 10% fetal bovine serum, penicillin/streptomycin, and mycostatin) was added to the monolayer of cells. After a 48-h incubation, the supernatant from cell monolayers was combined with defibrinated bovine blood and adenosine triphosphate (0.005 M) as a phagostimulant to create 7.3 to 8.3 log_10_ pfu/mL infectious blood meals through which mosquitoes fed using a Hemotek feeding system. Following feeding trials, mosquitoes were sorted, and fully engorged females were returned to cylindrical cages along with access to 10% sucrose solution and water and maintained in an incubator at 30 °C and a 14:10 hour light:dark photo regime.

### 2.3. Transmission Assays

To approximate the ability to transmit chikungunya virus, mosquitoes were deprived of sucrose for 24 h and then transferred to clear plastic tubes (height × diameter: 8 by 3 cm) along with an oviposition substrate. Each tube held a single female and was fitted with a mesh lid that had a piece of filter paper (1 cm diameter) or FTA card saturated with honey dyed blue. Cohorts of mosquitoes were tested for transmission at 5 to 6 and 12 to 13 days after ingesting chikungunya virus infectious blood. Mosquitoes were examined after 24 and 48 h following the start of the transmission assay using a flashlight to determine whether blue coloring could be visualized in the crop, an indicator that the mosquito fed and deposited saliva. Mosquitoes with blue coloring in their crop were then subject to forced salivation into the capillary tube to collect saliva expectorates using methods previously described [28]. Briefly, mosquitoes were cold-anesthetized, and their legs and wings were removed by dissection. The proboscis was inserted in capillary tubes loaded with immersion oil for 1 h to collect saliva. All mosquitoes were killed and stored at −80 °C upon completion of the transmission assay.

### 2.4. Viral RNA Isolation and qRT-PCR

Mosquitoes were individually dissected and the legs and saliva (both methods of collection) were subject to homogenization and centrifugation. The legs and pilter Paper were homogenized separately in 1.0 mL of 199 media. The saliva from mosquitoes collected in the capillary tubes was combined with 300 µL of media. A cell-free viral RNA isolate was obtained from a 160 µL mosquito sample of each tissue using a QIAamp Viral RNA Mini Kit (Thermo Fisher Scientific Inc.). Quantitative real-time polymerase chain reaction (qRT-PCR) using the CFX96 Real-Time PCR Detection System (Bio-Rad Laboratories, Hercules, CA, USA) was used to determine the presence and quantity of viral RNA in the test sample with the Superscript III One-Step qRT-PCR with a Platinum Taq kit by Invitrogen (Carlsbad, CA, USA) using the manufacturer’s recommendation [29]. Chikungunya virus primers were designed to target a nonstructural polyprotein gene common to both lineages (accession ID of transcript, KU365292.1) with the following sequences: forward, 5′-GTACGGAAGGTAAACTGGTATGG-3′: reverse, 5′TCCACCTCCCACTCCTTAAT-3′. The probe sequence was: 5′-/56-FAM/TGCAGAACCC ACCGAAAGGAAACT/3BHQ_1/-3′ (Integrated DNA Technologies, Coralville, IA). The program for qRT-PCR was as follows; 50 °C for 30 min, 94 °C for 2 min, 39 cycles at 94 °C for 10 s and 60 °C for 1 min, and lastly 50 °C for 30 s. A standard curve method was used to express the titer of chikungunya virus in the mosquito samples by comparing cDNA synthesis for a range of serial dilutions of chikungunya virus in parallel with plaque assays of the same dilutions of the virus, expressed as plaque-forming unit equivalents (pfue)/mL [30]). Testing the legs of mosquitoes provided us with an indicator of which mosquitoes had a disseminated infection. The transmission was calculated as the proportion of saliva infected mosquitoes from the total number of mosquitoes with infected legs.

### 2.5. Statistical Analyses

Two sections of analyses were performed. In the first part of the analyses, we were interested in analyzing how viral dissemination on harvested mosquito legs (log_10_ transformed), mosquito population (Brazil, Dominican Republic, and USA), and days post-infection (dpi, 2, 5 to 6, and 12 to 13) affected the presence or absence of CHIKV in mosquito saliva (a dichotomous dependent variable) and salivary viral titer (log_10_ transformed, a continuous dependent variable). In the second group of analyses, we were interested in comparing the efficacy of three well-established methods for CHIKV detection in mosquito saliva. We analyzed how the presence or absence of CHIKV in mosquito saliva and on salivary viral titer were affected by country of origin, days post-infection, and detection method (capillary tube, filter paper, and FTA card). Analyses were performed by fitting multivariable generalized linear models (GLM) with the binomial probability distribution (to model the effect of the independent variables on the presence or absence of CHIKV in mosquito saliva) and with the Gaussian probability distribution (to model the effect of the independent variables on the log_10_ transformed salivary viral titer). Separate analyses were performed for both used CHIKV genotypes (Asian and IOL) for *Ae. aegypti* and *Ae. albopictus*. The separate analyses were needed because of unbalanced categories in the independent variables. For the Asian genotype, country of origin for *Ae. aegypti* included Brazil, Dominican Republic, and the US; for *Ae. albopictus*, country of origin included Brazil and the US. For the IOL genotype, country of origin for *Ae. aegypti* included the Dominican Republic and the US; for *Ae. lbopictus*, country of origin included only the US. Detection methods for *Ae. aegypti* and *Ae. albopictus* from Brazil and USA included capillary tube, filter paper, and FTA cards. Detection methods used for the Asian genotype included all three methods, while for the IOL genotype only capillary tube and filter paper were included. Exploratory analyses were done by constructing contingency tables, figures and univariate logistic models were adjusted to analyze the overall relationship between the dependent variable and the independent variables. Likelihood-ratio Chi-square tests were used to test the significance of the sequential inclusion of the independent variables in binomial GLMs, while likelihood-ratio F tests were used to test the significance of the sequential inclusion of the independent variables in the Gaussian GLMs [31]. All analyses were done using R [32] and RStudio [33], with the library ggplot2 [34].

## 3. Results

### 3.1. Overall Results

**Figure 1:**
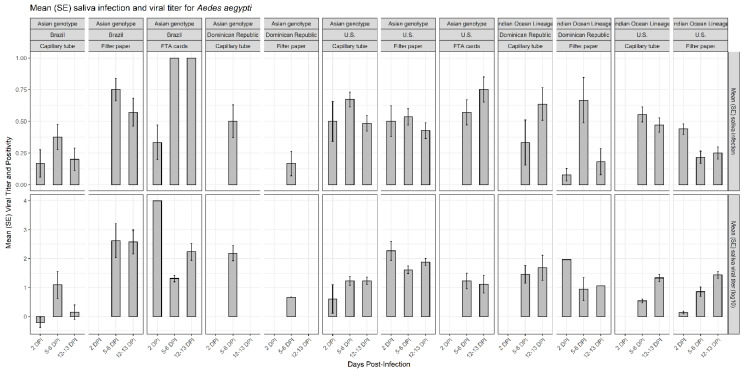
Mean (SE) proportion of saliva infection and viral titer (log_10_ pfue/mL) of *Ae. aegypti* females from Brazilian, Dominican Republic, and US populations, measured by three different methods (capillary tube, filter paper, and FTA card) at different days post-infection (DPI; 1 = 2 DPI, 2 = 5 to 6 DPI, 3 = 12 to 13 DPI), for two CHIKV genotypes (Asian and IOL).

**Figure 2:**
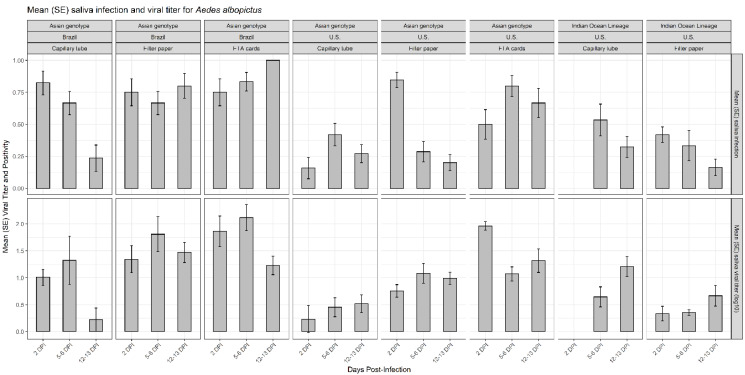
Mean (SE) proportion of saliva infection and viral titer (log_10_ pfue/mL) of *Ae. albopictus* from Brazilian and US populations, measured by three different methods (capillary tube, filter paper, and FTA card) at different days post-infection (DPI; 1 = 2 DPI, 2 = 5 to 6 DPI, 3 = 12 to 13 DPI), for two CHIKV genotypes (Asian and IOL).

Chikungunya virus infection rates were measured by the proportion of mosquitoes that had infected saliva from the total that presented blue coloring in their crop. A total of 1647 *Aedes* mosquitoes presented blue crops, indicating deposition of saliva on the substrate (filter paper, FTA card, capillary tube) following ingestion of an infectious blood meal and incubation. The 1046 total specimens of *Ae. aegypti* were composed of 168 individuals from Brazilian populations (with an overall positivity of 0.415 ± 0.0380 and a viral titer of 1.21 ± 0.159 log_10_ pfue/mL; mean ± SE), 101 from a Dominican Republic population (positivity of 0.338 ± 0.0471; viral titer of 1.56 ± 0.118 log_10_ pfue/mL) and 777 from US populations (positivity of 0.448 ± 0.0178; viral titer of 1.02 ± 0.0406 log_10_ pfue/mL). Of the 601 *Ae. albopictus*, 183 were from Brazilian populations (positivity of 0.648 ± 0.0353; viral titer of 1.22 ± 0.118 log_10_ pfue/mL) and 418 from US populations (positivity of 0.354 ± 0.0234; viral titer of 0.617± 0.0482 log_10_ pfue/mL) (Table 1, Figure 1 and Figure 2). For the CHIKV Asian genotype, FTA cards detected a higher proportion of infected saliva from females of *Ae. aegypti* (0.818 ± 0.0515 for the Brazilian population and 0.636 ± 0.0655 for the US population) and *Ae. albopictus* (0.833 ± 0.0477 for the Brazilian population and 0.667 ± 0.0609 for the US population). For the IOL genotype, the capillary tube detected the higher proportion of infected saliva for *Ae. aegypti* (0.529 ± 0.109 for the Dominican Republic population and 0.511 ± 0.0408 for the US population) and for *Ae. albopictus* (0.391 ± 0.0704 for the US population) (Table 1, Figure 1 and Figure 2).

### 3.2. Effects of Viral Dissemination, Country of Origin, and Days Post-Infection on CHIKV Salivary Positivity and CHIKV Viral Titer on Mosquito Saliva

**Asian genotype:** For *Ae. aegypti*, binomial GLM results showed a significant effect of viral dissemination on CHIKV salivary positivity (LR Chi-square test = 17.65, df = 1, *p* < 0.001), meaning that for each increase of 1 log_10_ pfue/mL of viral dissemination the odds of salivary infections increased by 49.00%. No significant effects of country of origin and days post-infection were observed (Table 2). Gaussian GLM results showed a significant positive effect of viral dissemination (LR F test = 16.7, df = 1, *p* < 0.001) and country of origin (LR F test = 7.648, df = 2, *p* < 0.05) on CHIKV viral titer on female saliva, meaning that for each increment of 1 log_10_ pfue/mL in CHIKV dissemination increased CHIKV saliva titer by 0.265 log_10_ pfue/mL while controlling by all other variables. Country effects showed that *Ae. aegypti* from the Dominican Republic had a CHIKV salivary viral titer 1.219 log_10_ pfue/mL higher than Brazilian mosquitos on average. No differences were observed when comparing the US population with Brazilian and when comparing the US population with the Dominican Republic (Table 2).

For *Ae. albopictus*, binomial GLM results showed that viral dissemination (LR Chi-square test = 9.765, df = 1, *p* < 0.01), country of origin (LR Chi-square test = 15.77, df = 1, *p* < 0.01), and days post-infection (LR Chi-square test = 18.51, df = 2, *p* < 0.001) were significant on CHIKV salivary positivity. For each increase of 1 log_10_ pfue/mL on viral dissemination the odds of saliva infection increased by 39.57%. The odds of *Ae. albopictus* from US presenting saliva infection were 68.57% lower than the Brazilian population. Finally, the odds of *Ae. albopictus* presenting salivary infection significantly decreases at 12 to 13 dpi when compared with 2 dpi (81.28% lower), and when comparing 12 to 13 dpi with 5 to 6 dpi (60.14% lower, Tukey HSD post-hoc comparison, *p* < 0.05; Table 2). Gaussian GLM results showed significant effects of viral dissemination (LR F test = 18.39, df = 1, *p* < 0.001) and country of origin (LR F test = 6.049, df = 1, *p* < 0.05) on CHIKV viral titer in the saliva of female *Ae. albopictus*. For each increase of 1 log_10_ pfue/mL of viral dissemination, an increase of 0.246 log_10_ pfue/mL in CHIKV saliva titer, and that *Ae. albopictus* of US origin had an average of 0.401 log_10_ pfue/mL lower viral titer than females from Brazil (Table 2).

**IOL genotype:** For *Ae. aegypti*, binomial GLM results showed a significant effect of viral dissemination on CHIKV salivary positivity (LR Chi-square test = 23.43, df = 1, *p* < 0.001), meaning that for each 1 log_10_ pfue/mL of viral dissemination the odds of salivary infections increase by 29.17%. No significant effect of country of origin and days post-infection were observed (Table 2). Gaussian GLM results showed a significant negative effect of country of origin (LR F test = 4.031, df = 1, *p* < 0.05) and positive effect of days post-infection (LR F test = 46.39, df = 1, *p* < 0.001) on CHIKV viral titer on female saliva. The US population of *Ae. aegypti* had a CHIKV salivary viral titer 0.454 log_10_ pfue/mL lower when compared with the Dominican Republic population. Salivary viral titer significantly increased with each dpi; 0.383 log_10_ pfue/mL at 5 to 6 dpi and 1.104 log_10_ pfue/mL at 12 to 13 dpi when comparing with 2 dpi, and 0.721 log_10_ pfue/mL when comparing 12 to 13 dpi with 5 to 6 dpi (Tukey HSD post-hoc comparison, *p* < 0.001) (Table 2).

For *Ae. albopictus*, binomial GLM results showed that neither viral dissemination nor days post-infection significantly affected CHIKV saliva positivity, although the latter effect was only marginally non-significant (*p* = 0.05001). Gaussian GLM results showed a significant effect of days post-infection (LR F test = 14.37, df = 1, *p* < 0.001), with an increasing salivary viral titer with each passing dpi; 0.509 log_10_ pfue/mL at 5 to 6 dpi and 0.841 log_10_ pfue/mL at 12 to 13 dpi when comparing with 2 dpi, but no differences when comparing 12 to 13 dpi with 5 to 6 dpi (Table 2).

### 3.3. Efficacy of Capillary Tube, Filter Paper, and FTA Cards for CHIKV Detection and CHIKV Viral Titer in Mosquito Saliva

**Asian genotype:** For *Ae. aegypti*, binomial GLM results showed significant effects of detection method (LR Chi-square test = 6.2483, df = 2, *p* < 0.05), country of origin (LR Chi-square test = 4.7541, df = 1, *p* < 0.05) and days post-infection (LR Chi-square test = 7.9379, df = 2, *p* < 0.05). The odds of detecting positive CHIKV saliva of *Ae. aegypti* using FTA cards is 3.375 times higher than using the capillary tube, but no difference was found when comparing filter paper with capillary tube and FTA cards with filter paper. The odds of *Ae. aegypti* from US presenting saliva infection are 81.72% higher than the Brazilian population. The odds of detecting saliva infection at 5 to 6 dpi was 2.207 times higher when compared with 2 dpi. No differences were found when comparing 12 to 13 dpi with 2 dpi and 12 to 13 dpi with 5 to 6 dpi (Table 3). Gaussian GLM results showed significant effects of country of origin (LR F test = 36.22, df = 1, *p* < 0.001) and days post-infection (LR F test = 122.0213, df = 2, p < 0.001), but no significant effect of detection method on CHIKV viral titer on female saliva. *Aedes aegypti* from the US had on average 0.699 log_10_ pfue/mL higher CHIKV viral titer than the Brazilian population. Salivary viral titer significantly increased with each dpi; 1.722 log_10_ pfue/mL and 1.639 log_10_ pfue/mL at 5 to 6 dpi and 12 to 13 dpi when compared with 2 dpi, respectively (Table 3).

For *Ae. albopictus*, binomial GLM results showed significant effects of detection method (LR Chi-square test = 8.3740, df = 2, *p* < 0.05), country of origin (LR Chi-square test = 15.9364, df = 1, *p* < 0.001) and days post-infection (LR Chi-square test = 10.7430, df = 2, *p* < 0.01). The odds of detecting positive CHIKV saliva of *Ae. albopictus* using FTA cards is 3.836 times higher than using capillary tube, but no difference was found when comparing filter paper with capillary tube and FTA cards with filter paper. The odds of *Ae. albopictus* from the US presenting saliva infection were 69.09% lower than the Brazilian population. The odds of detecting saliva infection at 12 to 13 dpi was 65.96% times lower when compared with 2 dpi (Table 3). Gaussian GLM results showed significant effect only of days post-infection (LR F test = 78.1183, df = 2, *p* < 0.001). Salivary viral titer significantly increased with each dpi; 1.332 log_10_ pfue/mL and 1.391 log_10_ pfue/mL at 5 to 6 dpi and 12 to 13 dpi when compared with 2 dpi, respectively (Table 3).

**IOL genotype:** For *Ae. aegypti*, binomial GLM results showed significant effects of detection method (LR Chi-square test = 22.9190, df = 1, *p* < 0.001) and days post-infection (LR Chi-square test = 8.7540, df = 2, *p* < 0.05). The odds of detecting saliva infection using filter paper was 69.04% lower than using a capillary tube. The odds of detecting saliva infection at 5 to 6 dpi was 48.91% lower when comparing to 2 dpi, and 54.36% lower when comparing 12 to 13 dpi with 2 dpi (Table 3). Gaussian GLM results showed significant effects of country of origin (LR F test = 7.2855, df = 1, *p* < 0.01) and days post-infection (LR F test = 13.3724, df = 2, *p* < 0.01), but no significant effect of detection method on CHIKV viral titer on female saliva. *Aedes aegypti* from the US had on average 0.807 log_10_ pfue/mL lower CHIKV viral titer than the Dominican Republic population. Salivary viral titer significantly increased at 12 to 13 dpi when compared with 2 dpi (0.82452 log_10_ pfue/mL) and when comparing 12 to 13 dpi with 5 to 6 dpi (0.613 log_10_ pfue/mL) (Table 3).

For *Ae. albopictus*, binomial GLM results showed a significant effect for days post-infection (LR Chi-square test = 8.5277, df = 2, *p* < 0.05). The odds of detecting saliva infection at 12 to 13 dpi was 72.84% lower when comparing to 2 dpi (Table 3). Gaussian GLM results showed significant effects for days post-infection (LR F test = 16.6294, df = 2, *p* < 0.001), with an increasing CHIKV salivary viral titer with each passing dpi: 1.188 log_10_ pfue/mL higher at 5 to 6 dpi when comparing with 2 dpi, and 1.341 log_10_ pfue/mL higher at 12 to 13 dpi when comparing with 2 dpi (Table 3).

## 4. Discussion

Vector competence is a phenotypic parameter defined as the ability of the vector to become infected, replicate, and transmit a pathogen [21,35,36]. Vector competence measured in terms of dissemination and/or infection rate is often expressed in the percentage of engorged females with virus detected in the head (as a proxy for the salivary glands). However, a complete assessment of vector competence necessitates the detection of arbovirus infection of saliva which quantifies infectious mosquitoes that are capable of transmitting the arbovirus via a bite of a vertebrate host. There are different direct and indirect available methods to estimate the amount of virus inoculated during transmission in a female mosquito’s saliva. Of the direct methods, included are the detection of the virus in hanging drops of blood fed upon by mosquitoes, detection of the virus in the vertebrate host’s tissue right after mosquito feeding, detection of the virus in blood-agar fed upon by mosquitoes, and detection of the virus in fluids such as immersion oil after mosquito salivation [19,22]. Saliva collection via the capillary tube method has been used in laboratory infection experiments to capture saliva expectorates from live female mosquitoes, including CHIKV [21,22,26]. However, the amount of saliva collected and the associated amount of virus may differ depending on the collection method and exposure time. In the present study, we compared the relative efficacy of three different methods: capillary tube, filter paper and FTA cards for detection of two CHIKV genotypes in the saliva expectorate of two putative vectors species, *Ae. aegypti* and *Ae. albopictus*.

General analyses for *Ae. aegypti* and *Ae. albopictus*, for the Asian genotype, showed that FTA cards was the method that detected the higher proportion of infected females, while for the IOL genotype it was the capillary tube for both species. Further studies could investigate differences in rates of viral RNA degradation by detection method, while also accounting for population origin and days post-infection, which would make it possible to measure the effect of phenotypic differences in vector competence [20,26,37]. For example, the capillary tube collects saliva at a 1-h interval in laboratory conditions, possibly minimizing RNA degradation [19]. In contrast, the filter paper and FTA card take a longer interval for proper collection (one to two days) [21]. Also, FTA cards include chemistry to inactivate and preserve RNA, whereas filter paper has no added features of RNA preservation [21,38,39]. The latter observation may be important in considering that there are typically low amounts of virus in mosquito saliva, thus approaching the limit of detection, especially in instances when some viral RNA degradation has occurred. In fact, our results showed a diverse profile of viral titers by country of origin and detection method for both CHIKV genotypes. For instance, mean viral titers for *Ae. aegypti* ranged from 0.509 to 2.596 log_10_ pfue/mL for the Asian genotype, and from 0.498 to 1.630 log_10_ pfue/mL for the IOL genotype depending on the population and detection method used. For *Ae. albopictus*, viral titers ranged from 0.420 to 1.886 log_10_ pfue/mL for the Asian genotype and from 0.318 to 0.958 log_10_ pfue/mL for the IOL genotype. Filter paper was, in general, the detection method responsible for lower viral titers, with the exception of Brazilian *Ae. aegypti*, which presented the capillary tube as the detection method with the lower detected viral titer.

We used quantitative RT-PCR for the detection of chikungunya virus in mosquito samples. Although this molecular approach is common, it should be interpreted with caution. The expression of plaque-forming unit equivalents (pfue)/mL does not distinguish between viable or non-viable virions. That is, tissue culture techniques (e.g., plaque assay and TCID50) detect live virus only. In the context of this study, quantitative RT-PCR may over-estimate the amount of virus present in saliva. However, this is not a major issue since the qRT-PCR is standardized with a plaque assay approach.

Smith et al. [19] discussed methodological questions regarding the different routes for mosquito infection and the use of capillary tubes, including immersion oil and fetal bovine serum-FBS for saliva collection and to measure viral expectoration. The authors also pose a question that we further develop here on whether the amount of virus a mosquito salivates into a capillary tube accurately reflects the amount transmitted during blood-feeding on a vertebrate host as assessed with capillary tubes, filter paper, or FTA cards. These authors compared three traditional artificial transmission methods, using the Venezuelan equine encephalitis virus (VEEV) and *Ae. albopictus* and *Ochlerotatus taeniorhynchus* mosquitoes. Their results showed that both mosquito species and the infection route used affected the amount of virus detected in the saliva after a 10-day incubation period. Median titers of virus detected in the saliva of *Ae. albopictus* and *Oc. taeniorhynchus* mosquitoes ranged from 0.2 to 1.1 log_10_ (mean 0.7 to 1.4 log_10_) and 0.2 to 3.2 log_10_ (mean 1.0 to 3.6 log_10_) plaque-forming units, respectively. The questions posed by Smith et al. [19] can be answered with further experiments, especially considering the role of not only different mosquito species and arboviruses but also with different geographic origins. Guedes et al. [40] performed mosquito vector competence assays under laboratory conditions, comparing both *Ae. aegypti* and *Culex quinquefasciatus* using different virus doses. The authors showed that both *Ae. aegypti* and *Cx. quinquefasciatus* can be experimentally infected by ZIKV even at low doses 10^4^ pfu/mL and can subsequently expectorate ZIKV in their saliva. Also, another study evaluated experimentally the effect of variable temperature regimes on disseminated infection and saliva infection of the *Aedes* mosquitoes [41]. These authors found evidence that the number of mosquitoes with disseminated infection, but not saliva infection, in *Ae. aegypti* and *Ae. albopictus* was influenced by an interaction of the geographic origin of the mosquito and temperature regime, suggesting small scale geographic variation of CHIKV infection in potential *Aedes* vectors.

Coffey et al. [42] summarized numerous CHIKV infections in *Ae. aegypti* and *Ae. albopictus*, showing that infection, dissemination, and transmission rates of both vectors are dependent on the geographic sources of mosquito populations. Vega-Rúa et al. [20] tested 35 different American populations of *Ae. aegypti* and *Ae. albopictus* for three CHIKV genotypes, including mosquitoes from Brazil and Florida but not from the Dominican Republic. The study showed that all 35 populations of both *Aedes* vectors from 10 different countries were susceptible to CHIKV infection by the three tested genotypes. However, CHIKV transmission efficiency was highly heterogeneous, ranging from 11.1% to 96.7%. Alto et al. [21] evaluated the potential for sustained local transmission of CHIKV in Florida by testing whether the local population of *Ae. aegypti* and *Ae. albopictus* exhibited differences in their susceptibility to CHIKV infection and transmission using IOL and Asian genotypes in laboratory experiments. Results showed that both *Aedes* species displayed susceptibility to infection, rapid viral dissemination into the hemocoel, and transmission for both emergent lineages of CHIKV. *Aedes aegypti* originating from the Dominican Republic had lower viral dissemination and transmission rates for IOL and Asian genotypes when compared to Florida vectors. The authors also identified small-scale geographic variation in vector competence among both species that might contribute to regional differences in the risk of CHIKV transmission in Florida. In our study, we also found a heterogeneous profile of significance for the country of origin of mosquitoes in binomial models that tested the effects of viral dissemination. For the Asian genotype, we found that *Ae. albopictus* of US origin had lower odds of displaying saliva infection than from Brazilian origin [26]. The lack of significance for *Ae. aegypti* shows that Brazilian, Dominican Republic, and US mosquitoes are equally susceptible to saliva infection by this genotype. Thus, our results corroborate previous studies that show the heterogeneous profile of vector competence for CHIKV for both species in the Americas.

For the Asian genotype, binomial models for *Ae. aegypti* showed that FTA cards were the more effective of the employed methods to detect CHIKV positive saliva from females when compared to capillary tubes and filter papers, controlling by all other variables. Post-hoc comparisons showed that, when comparing FTA cards with capillary tubes, the odds were 3.375 higher on average, and when compared to filter paper, the odds were only marginally significant, 3.344 higher in average (*p* = 0.0603), when controlling by all other variables. The significance of country of origin and days post-infection reveals a complex pattern of CHIKV infection in *Ae. aegypti* from Brazil, Dominican Republic, and US populations [20,21,26]. Binomial models for the Asian genotype for *Ae. albopictus* showed a significant effect of the detection method, with FTA cards significant when compared to capillary tubes (with odds of 3.836) and no significance when compared to filter paper. Despite the lack of significance when comparing FTA cards with filter paper, it is possible to point out FTA cards as a more effective method to detect positive saliva for the Asian genotype.

The strategy of viral RNA detection from FTA cards has been employed in previous studies for arbovirus surveillance [23,40,43]. The use of FTA cards as a tool for arbovirus surveillance is being increasingly described as successful in field studies, being a faster detection method than laboratory-based ones, like the use of capillary tubes or detection in macerates of field-collected mosquitoes. Guedes et al. [40] exposed mosquitoes to honey-soaked FTA cards placed on the top of the cages to collect *Ae. aegypti* and *Cx. quinquefasciatus* saliva. These authors successfully detected ZIKV RNA copies in cards from these mosquitoes, demonstrating that, in addition to being susceptible to ZIKV infection and allowing virus replication in the salivary glands, both species were found capable of effectively transmitting ZIKV. Flies et al. [42] were able to detect several arboviruses by using modified light traps with honey cards in a field surveillance program of encephalitis vectors in Southern Australia. In Northern Australia, van den Hurk et al. [44] were able to detect arbovirus during field collections of mosquitoes by using sugar-baited nucleic acid preservation cards in 13.9% of the traps during the whole study period. Burkett-Cadena et al. [45] employed different collection methods for mosquitoes and used sugar-impregnated nucleic-acid preserving substrates and sentinel chicken program in Florida. Although the sentinel chicken provided a higher number of arbovirus detections than the sugar-impregnated substrates, they point out the need to optimize the traps to use the later method, which would provide an easy to use field method to detect arboviruses.

## 5. Conclusions

Our results for *Ae. aegypti* and *Ae. albopictus* introduce high-throughput alternatives for capturing mosquito saliva for arbovirus surveillance and showed an important significant effect of the detection method and viral titer on the odds of a female being positive for CHIKV saliva infection. There was an important effect of viral dissemination on harvested legs with the Asian genotype on increasing the odds of a female having infected saliva and higher viral titers, but not for the IOL genotype. Results for the Asian genotype for *Ae. aegypti* showed that FTA cards were more effective to detect positive CHIKV saliva from females when compared to capillary tubes, and no difference was observed when comparing filter paper with capillary tubes. No effects of the detection method were observed for detecting higher viral titer in infected saliva from females for both genotypes.

## Figures and Tables

**Table 1:** Descriptive statistics of the proportion of positive saliva infection (mean and SE) and viral titer (log_10_ pfue/mL, mean, and SE) for two CHIKV genotypes (Asian and IOL) for *Ae. aegypti* and *Ae. albopictus* from three different countries (Brazil, Dominican Republic, and the US) and using three collection methods (capillary tube, filter paper, and FTA card).

Genotype	Species	Country	Detection Method	Positivity (Mean)	Positivity (SE)	Viral Titer (Mean)	Viral Titer (SE)
Asian	*Aedes aegypti*	Brazil	Capillary tube	0.2679	0.0592	0.5086	0.2369
			Filter paper	0.6667	0.063	2.5959	0.3174
			FTA cards	0.8182	0.0515	2.0199	0.1644
		Dominican Republic	Capillary tube	0.5	0.1291	2.1854	0.2619
			Filter paper	0.1667	0.0962	0.6653	0.0038
		US	Capillary tube	0.5725	0.0423	1.1672	0.0998
			Filter paper	0.4808	0.0416	1.8146	0.0875
			FTA cards	0.6364	0.0655	1.1783	0.1713
	*Aedes albopictus*	Brazil	Capillary tube	0.5902	0.063	0.9792	0.2221
			Filter paper	0.7333	0.0566	1.5888	0.1563
			FTA cards	0.8333	0.0477	1.8855	0.1413
		US	Capillary tube	0.2989	0.048	0.4204	0.1068
			Filter paper	0.3846	0.0473	0.8981	0.0738
			FTA cards	0.6667	0.0609	1.3243	0.0876
IOL	*Aedes aegypti*	Dominican Republic	Capillary tube	0.5294	0.1089	1.6296	0.3121
			Filter paper	0.2333	0.0598	1.1342	0.1291
		US	Capillary tube	0.5115	0.0408	0.9062	0.0778
			Filter paper	0.3346	0.0276	0.5059	0.0569
	*Aedes albopictus*	US	Capillary tube	0.3913	0.0704	0.9577	0.1367
			Filter paper	0.3333	0.0443	0.384	0.0949

**Table 2:** Estimated effects and 95% confidence interval of viral dissemination, country of origin, and days post-infection for the Asian and IOL CHIKV genotypes for both *Ae. aegypti* and *Ae. albopictus*, as measured by in the binomial and Gaussian generalized linear models.

Species		Asian Genotype			IOL Genotype		
***Aedes aegypti***	**Binomial model for CHIKV saliva infection**						
	Effects	Estimate	SE	CI95	Estimate	SE	CI95
	Intercept	**0.176**	**0.4814**	**[0.065, 0.436]**	**0.336**	**0.3798**	**[0.156, 0.697]**
	Viral Dissemination (legs)	**1.49**	**0.1**	**[1.232, 1.826]**	**1.292**	**0.05409**	**[1.163, 1.438]**
	Country: Dom. Republic	0.874	0.5546	[0.288, 2.582]	(not included in the model)		
	Country: US	1.221	0.2773	[0.708, 2.107]	1.521	0.3443	[0.785, 3.051]
	5–6 dpi	1.261	0.4211	[0.547, 2.881]	0.87	0.2503	[0.532, 1.421]
	12–13 dpi	0.727	0.4148	[0.32, 1.644]	0.66	0.2535	[0.4, 1.082]
	**Gaussian model for CHIKV viral titer in saliva**						
	Intercept	−0.1562	0.3079	[−0.76, 0.447]	**0.5368**	**0.2579**	**[0.031, 1.042]**
	Viral Dissemination (legs)	**0.265**	**0.06485**	**[0.138, 0.392]**	0.04749	0.0334	[−0.018, 0.113]
	Country: Dom. Republic	**1.219**	**0.4636**	**[0.311, 2.128]**	(not included in the model)		
	Country: US	0.2706	0.1868	[−0.095, 0.637]	**−0.4543**	**0.2263**	**[−0.898, −0.011]**
	5–6 dpi	0.02319	0.2652	[−0.497, 0.543]	**0.3832**	**0.1552**	**[0.079, 0.687]**
	12–13 dpi	0.1753	0.2709	[−0.356, 0.706]	**1.104**	**0.164**	**[0.783, 1.426]**
***Aedes albopictus***	**Binomial model for CHIKV saliva infection**						
	Intercept	1.278	0.4299	[0.549, 2.982]	0.653	0.3225	[0.342, 1.219]
	Viral Dissemination (legs)	**1.396**	**0.1115**	**[1.129, 1.751]**	1.035	0.1067	[0.841, 1.281]
	Country: US	**0.314**	**0.2973**	**[0.174, 0.559]**	(not included in the model)		
	5–6 dpi	0.47	0.4101	[0.205, 1.03]	1.057	0.4683	[0.417, 2.645]
	12–13 dpi	**0.187**	**0.4255**	**[0.079, 0.421]**	**0.41**	**0.4248**	**[0.175, 0.931]**
	**Gaussian model for CHIKV viral titer in saliva**						
	Intercept	0.3357	0.2489	[−0.152, 0.823]	0.2326	0.1381	[−0.038, 0.503]
	Viral Dissemination (legs)	**0.2461**	**0.05738**	**[0.134, 0.359]**	−0.0664	0.05264	[−0.17, 0.037]
	Country: US	**−0.4006**	**0.1629**	**[−0.72, −0.081]**	(not included in the model)		
	5–6 dpi	−0.1732	0.2037	[−0.573, 0.226]	**0.5093**	**0.2159**	**[0.086, 0.932]**
	12–13 dpi	−0.3732	0.2208	[−0.806, 0.06]	**0.8416**	**0.225**	**[0.401, 1.283]**

Binomial model estimates and confidence intervals are presented as odds-ratio (calculated as the exponential of the estimated effect). Bold entries indicate statistical significance.

**Table 3:** Estimated effects and 95% confidence interval of detection method, country of origin, and days post-infection for the Asian and IOL CHIKV genotypes for both *Ae. aegypti* and *Ae. albopictus*, as measured by in the binomial and Gaussian generalized linear models.

Species		Asian Genotype			IOL Genotype		
***Aedes aegypti***	**Binomial model for CHIKV saliva infection**						
	Effects	Estimate	SE	CI95	Estimate	SE	CI95
	Intercept	**0.388**	**0.3891**	**[0.176, 0.818]**	1.791	0.4275	[0.769, 4.139]
	Method: Filter Paper	1.009	0.2433	[0.626, 1.626]	**0.31**	**0.2511**	**[0.188, 0.503]**
	Method: FTA Card	**3.375**	**0.5205**	**[1.275, 10.12]**	*(not included in the model)*		
	Country: US	**1.817**	**0.2767**	**[1.062, 3.151]**	1.255	0.3345	[0.66, 2.468]
	5–6 dpi	**2.207**	**0.3761**	**[1.066, 4.694]**	**0.511**	**0.2848**	**[0.29, 0.888]**
	12–13 dpi	1.232	0.3773	[0.592, 2.623]	**0.456**	**0.2828**	**[0.26, 0.79]**
	**Gaussian model for CHIKV viral titer in saliva**						
	Intercept	**2.112**	**0.1723**	**[1.774, 2.45]**	**2.041**	**0.3863**	**[1.284, 2.798]**
	Method: Filter Paper	−0.02606	0.1221	[−0.265, 0.213]	7.09 × 10^16^	0.2202	[−0.431, 0.431]
	Method: FTA Card	−0.01022	0.1454	[−0.295, 0.275]	*(not included in the model)*		
	Country: US	**0.6995**	**0.1162**	**[0.472, 0.927]**	**−0.807**	**0.299**	**[−1.393, −0.221]**
	5–6 dpi	**1.722**	**0.1623**	**[1.404, 2.04]**	0.2116	0.2527	[−0.284, 0.707]
	12–13 dpi	**1.64**	**0.1648**	**[1.317, 1.963]**	**0.8245**	**0.2474**	**[0.34, 1.309]**
***Aedes albopictus***	**Binomial model for CHIKV saliva infection**						
	Intercept	**2.219**	**0.3419**	**[1.148, 4.412]**	1.732	0.5437	[0.604, 5.145]
	Method: Filter Paper	1.662	0.3253	[0.881, 3.168]	0.417	0.479	[0.158, 1.048]
	Method: FTA Card	**3.835**	**0.5279**	**[1.427, 11.641]**	*(not included in the model)*		
	Country: US	**0.309**	**0.3004**	**[0.17, 0.553]**			
	5–6 dpi	0.799	0.3555	[0.395, 1.6]	0.675	0.5224	[0.235, 1.849]
	12–13 dpi	**0.34**	**0.3656**	**[0.164, 0.692]**	**0.272**	**0.4935**	**[0.098, 0.69]**
	**Gaussian model for CHIKV viral titer in saliva**						
	Intercept	**2.785**	**0.1863**	**[2.419, 3.15]**	**1.744**	**0.3994**	**[0.961, 2.527]**
	Method: Filter Paper	0.02291	0.164	[−0.298, 0.344]	2.54 × 10^15^	0.3411	[−0.669, 0.669]
	Method: FTA Card	−0.1018	0.1774	[−0.45, 0.246]	*(not included in the model)*		
	Country: US	−0.2115	0.1418	[−0.489, 0.066]			
	5–6 dpi	**1.332**	**0.1717**	**[0.996, 1.669]**	**1.188**	**0.4018**	**[0.4, 1.976]**
	12–13 dpi	**1.391**	**0.1781**	**[1.042, 1.74]**	**1.341**	**0.3398**	**[0.675, 2.007]**

Binomial model estimates and confidence intervals are presented as odds-ratios (calculated as the exponential of the estimated effect). Bold entries indicate statistical significance (*p* < 0.05).
